# Competitive Exclusion and Metabolic Dependency among Microorganisms Structure the Cellulose Economy of an Agricultural Soil

**DOI:** 10.1128/mBio.03099-20

**Published:** 2021-01-05

**Authors:** Roland C. Wilhelm, Charles Pepe-Ranney, Pamela Weisenhorn, Mary Lipton, Daniel H. Buckley

**Affiliations:** a School of Integrative Plant Sciences, Cornell University, Ithaca, New York, USA; b Biosciences Division, Argonne National Laboratory, Lemont, Illinois, USA; c Environmental Molecular Sciences Laboratory, Pacific Northwest National Lab, Richland, Washington, USA; University of California, Irvine

**Keywords:** decomposition, ecological trade-offs, metagenomics, stable isotope probing, metaproteomics, metabolic dependency, competitive exclusion, surface ecology, soil carbon cycling, ecogenomics

## Abstract

Microorganisms that degrade cellulose utilize extracellular reactions that yield free by-products which can promote interactions with noncellulolytic organisms. We hypothesized that these interactions determine the ecological and physiological traits governing the fate of cellulosic carbon (C) in soil. We performed comparative genomics with genome bins from a shotgun metagenomic-stable isotope probing experiment to characterize the attributes of cellulolytic and noncellulolytic taxa accessing ^13^C from cellulose. We hypothesized that cellulolytic taxa would exhibit competitive traits that limit access, while noncellulolytic taxa would display greater metabolic dependency, such as signatures of adaptive gene loss. We tested our hypotheses by evaluating genomic traits indicative of competitive exclusion or metabolic dependency, such as antibiotic production, growth rate, surface attachment, biomass degrading potential, and auxotrophy. The most ^13^C-enriched taxa were cellulolytic *Cellvibrio* (*Gammaproteobacteria*) and *Chaetomium* (*Ascomycota*), which exhibited a strategy of self-sufficiency (prototrophy), rapid growth, and competitive exclusion via antibiotic production. Auxotrophy was more prevalent in cellulolytic *Actinobacteria* than in cellulolytic *Proteobacteria*, demonstrating differences in dependency among cellulose degraders. Noncellulolytic taxa that accessed ^13^C from cellulose (*Planctomycetales*, *Verrucomicrobia*, and *Vampirovibrionales*) were also more dependent, as indicated by patterns of auxotrophy and ^13^C labeling (i.e., partial labeling or labeling at later stages). Major ^13^C-labeled cellulolytic microbes (e.g., *Sorangium, Actinomycetales, Rhizobiales*, and *Caulobacteraceae*) possessed adaptations for surface colonization (e.g., gliding motility, hyphae, attachment structures) signifying the importance of surface ecology in decomposing particulate organic matter. Our results demonstrated that access to cellulosic C was accompanied by ecological trade-offs characterized by differing degrees of metabolic dependency and competitive exclusion.

## INTRODUCTION

Cellulose is a major structural component of plant biomass and serves as a resource for productive and diverse soil microorganisms (1). Since cellulose is insoluble and highly crystalline, it cannot be transported across cell membranes. Hence, microorganisms rely on extracellular reactions to digest its fibers into soluble by-products for cell metabolism. Due to the structural complexity of lignocellulose, cellulose degradation is facilitated by synergistic interactions between diverse enzymes that differ in specific activity (2, 3). Physiological traits also influence the deconstruction of cellulose fibers, such as those linked to surface colonization like hyphal growth by members of fungi and *Actinobacteria* (4, 5), gliding motility in *Bacteroidetes* (6), and the formation of cellulosomes by many anaerobes (7, 8). These circumstances predispose microorganisms involved in cellulose degradation to metabolic, spatial, and ecological interactions. However, due to a reliance on isolation and coculturing, the nature of ecological interactions within cellulose-degrading consortia remain poorly understood, despite evidence of their occurrence in amplicon-based stable-isotope probing (SIP) studies (9–18). Shotgun metagenomics coupled to DNA-SIP now provides the capacity to compare the ecogenomic traits of diverse microorganisms that participate directly in the cellulose economy as it occurs in soil.

The extracellular nature of cellulose degradation creates conditions where the fitness of individuals is contingent on both competition and facilitation. Competition for cellulose and its degradation products impose fitness costs on cellulolytic organisms, promoting antagonistic interactions (19–21). However, facilitation by commensal and mutualistic partners enhances degradation rates to the benefit of cellulolytic organisms (22). Many cellulolytic microbes have close relatives lacking in endoglucanases, suggesting adaptive benefits from the gain or loss of these genes (23). The beneficiaries of community metabolism should be expected to shed energetically costly traits, resulting in adaptive gene loss and evolution of metabolic dependency (24). For example, noncellulolytic bacteria can complement the metabolic functions of cellulolytic bacteria *in vitro*, through complementary catabolism (25, 26), vitamin biosynthesis (27), amino acid biosynthesis (28), or biosynthesis of other essential metabolites (29). Such metabolic dependency can occur through specific, tightly coupled interactions, such as syntrophic partnerships, or via loosely coupled and nonspecific dependencies, such as a reliance on metabolic by-products or the mortality of community members (30, 31). We expect the anabolic and catabolic by-products of cellulolytic microbes to structure trophic interactions (i.e., the cellulose economy) and the fate of cellulosic carbon in soil.

Shotgun metagenomics and the recovery of metagenome-assembled genomes (MAGs) provide a cultivation-independent means of performing comparative genomics to study the phylogenetic and functional characteristics of microbial communities. This approach has been used to identify ecogenomic traits (32, 33) and to study metabolic dependency in environmental populations (34). By coupling this approach with DNA-SIP, one can distinguish MAGs from organisms that assimilate ^13^C from labeled substrates by separating and sequencing the ^13^C-enriched DNA (see Fig. S1 at https://osf.io/tb3n4/). Metagenomic SIP proved effective in resolving traits of cellulolytic and lignolytic populations in forest soil by improving MAG assembly (14). DNA-SIP can be used to estimate the degree of ^13^C labeling of individual MAGs by measuring the change in buoyant density across the CsCl gradient (35, 36). This gradient-resolved approach offers the capacity to distinguish between highly ^13^C-enriched DNA, corresponding to taxa with primary access to cellulosic C, less ^13^C-enriched DNA, corresponding to microbes with peripheral access to cellulosic C, and unenriched DNA from the broader soil community. Information about genomic content coupled to the degree of access to cellulosic C can be used as evidence of ecological trade-offs, such as traits of metabolic dependency (auxotrophy) or antibiotic production, in members of the cellulose economy.

We conducted a metagenomic SIP experiment with ^13^C-labeled cellulose to test hypotheses about the ecological trade-offs occurring in microbes that access carbon during cellulose degradation. We used comparative genomics to identify features of genome bins grouped by their degree of ^13^C enrichment (according to gradient-resolved SIP) and competency for cellulose degradation. We expected that cellulolytic microbes would be enriched in secondary metabolite gene clusters (SM), such as those that synthesize antibiotics, to control access to community resources. We further hypothesized that ^13^C-enriched, noncellulolytic microbes would exhibit signatures of metabolic dependency based on the degree of auxotrophy and/or capacity for degrading microbial necromass (i.e., numbers of genes encoding nucleases, peptidases, chitinases, and other hydrolytic enzymes). We expected early colonizers of cellulose, as identified in a companion study (11), to depend less on the products of community metabolism than late colonists, evidenced by degree of auxotrophy. We also identified traits related to surface colonization, which we anticipated would facilitate interactions on insoluble fibers (37). Results were validated against reference genomes and with metaproteomics to confirm gene expression by target groups. This study aims to evaluate the ecological trade-offs among members of the cellulose economy and to propose a framework for understanding community dynamics responsible for cellulose decomposition in soils.

## RESULTS

### Overview of metagenome assembly and designation of ^13^C labeling.

Initially, we performed a DNA-SIP experiment with [^13^C]cellulose to evaluate the temporal dynamics of decomposition over a 30-day period, profiled using 16S rRNA gene amplicon sequencing as previously described (11). Here, we performed gradient-resolved DNA-SIP using the ^13^C-labeled DNA at day 30 to discern differences in the degree of ^13^C incorporation and compare populations based on their relationships to cellulosic C. Deep sequencing was performed on a single gradient-resolved sample to maximize the recovery of genomic information from all members of the cellulose economy to improve our capacity to perform comparative genomics. Comparative genomics was performed in parallel on reference genomes sourced from the NCBI based on similarity to the full-length 16S rRNA gene.

**FIG 1:**
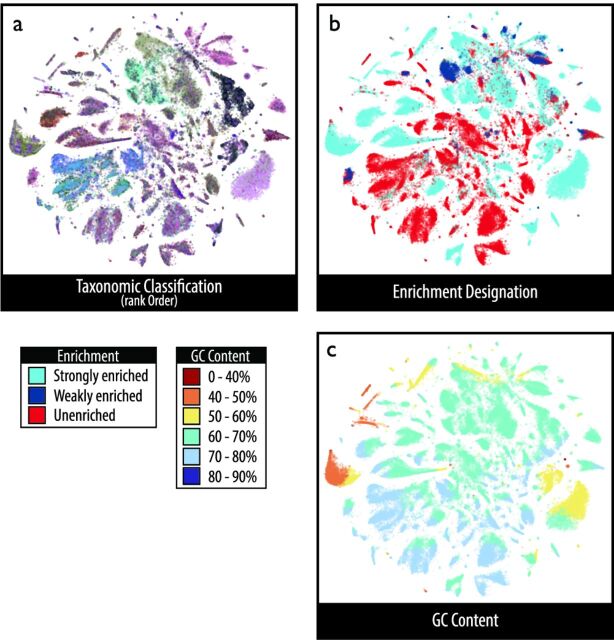
A visual representation of the whole metagenomic assembly reveals the clustering of contigs with respect to taxonomic classification (a), ^13^C enrichment designation (b), and genome GC content (c). Contigs (>3.5 kb) were clustered by pentanucleotide frequency using the t-SNE multidimension reduction algorithm. This figure reveals the correspondence between taxonomic classification and ^13^C enrichment status (a versus b) and that enrichment status was not correlated with GC content (b versus c). An interactive html version in the supplementary data package at https://osf.io/tb3n4/ allows for detailed exploration of taxonomic annotations for all contigs.

Shotgun metagenomic sequencing of ^13^C-labeled DNA recovered a total of 1.1 billion reads after quality filtering, estimated by nonpareil (38) to cover 80% of genomic diversity in the DNA pool. Metagenome assembly produced a total of 356,131 contigs greater than 2.5 kb, amounting to a total length of 1.8 Gb (∼230 genomes of 8 Mb). The degree of ^13^C enrichment was estimated for each contig by comparing the CsCl gradient profile to simulated natural abundance profiles (see Fig. S2 at https://osf.io/tb3n4/). More than half of all contigs were designated either strongly or weakly ^13^C enriched (total length, 921 Mb), while the remainder were from genomes of abundant soil taxa that lacked evidence of ^13^C labeling (766 Mb). The occurrence of unlabeled DNA in heavier fractions is common in SIP experiments, primarily reflecting differences in the migration of DNA fragments across the gradient based on G+C content (39). Contigs clustered into coherent sets according to pentanucleotide frequency which grouped by patterns of ^13^C labeling and taxonomy (Fig. 1a and b), and not average G+C content (Fig. 1c). The random forest model used to predict enrichment status had an overall accuracy of 89.1% with high sensitivity and specificity for both strongly enriched (98.3% and 86.4%, respectively) and unenriched (100% and 93%) contigs (see details in the supplementary methods and Table S1 at https://osf.io/tb3n4/).

### Phylobin characteristics.

**FIG 2:**
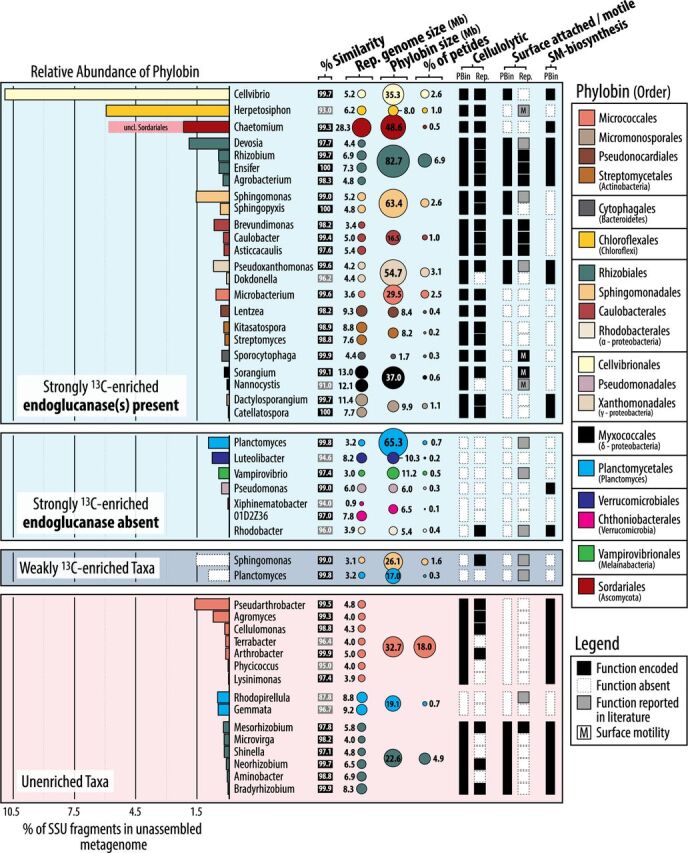
Members of the cellulose-degrading consortium were defined by their taxonomy and functional capabilities encoded in metagenome-assembled phylobins (PBin) and representative genomes (Rep). Phylobins were categorized by their ^13^C enrichment and cellulolytic capacity and ranked along the *y* axis by the relative abundance of SSU rRNA gene fragments recovered (indicated by bar plots). Representative genomes were identified according to similarity of full-length 16S rRNA genes (column 1) and were grouped with their respective phylobins. Representative genomes with less than 97% similarity to a phylobin 16S rRNA gene are shaded in gray. Several phylobins were comprised of genomes from multiple genera, and the size of each (in megabases) and the percentage of peptides assigned to each phylobin are provided. The remaining columns show the presence/absence of genes for endoglucanases or those involved in surface attachment, surface motility (M), and secondary metabolite (SM) production. Boxes are shaded gray if a member of that genus reportedly possesses the ability for attachment or surface motility. Secondary metabolite production is designated if peptides corresponding to antimicrobial gene clusters were detected in the metaproteome. Only the most abundant “unenriched” phylobins are shown (for a full overview, see Table S4 at https://osf.io/tb3n4/).

Contigs were grouped into phylobins based on ^13^C-labeling status and taxonomic classification (rank at the level of order). The large size of many phylobins can be attributed to natural pangenomic diversity (intraspecies) and to the grouping of mixed populations by order (interspecies), as revealed by the analysis of single-copy genes and single nucleotide polymorphisms per single-copy gene (see details in supplementary methods at https://osf.io/tb3n4/). A total of 47, 2, and 46 phylobins greater than 1 Mb were produced from strongly, weakly, and unenriched contig sets, respectively (see Table S2 at https://osf.io/tb3n4/). Of the 95 total phylobins, 38 were deemed high quality (>75% completeness) and were divided into four categories based on enrichment status and cellulolytic potential (inferred from the presence of endoglucanases): strongly ^13^C enriched and cellulolytic (*n*_strong_ = 12), strongly ^13^C enriched and noncellulolytic (*n*_strong_ = 8), weakly ^13^C enriched and noncellulolytic (*n*_weak_ = 2), and unenriched (*n *= 16). Each phylobin represents a genomic-ecological unit, rather than an individual genome, encompassing genomes from at most four to seven genera for enriched and unenriched phylobins, respectively, based on the diversity of assembled full-length 16S rRNA genes (Fig. 2). Both phylobins and representative reference genomes from ^13^C-enriched cellulolytic taxa were larger (μ_PhyBin_ = 24.7 Mb; μ_Rep_ = 7.1 Mb) than those from ^13^C-enriched noncellulolytic taxa (μ_PhyBin_ = 12.5 Mb; μ_Rep_ = 5.2 Mb; Wilcoxon test, *P ≤ *0.05) and unenriched taxa (μ_PhyBin_ = 11.4 Mb; μ_Rep_ = 5.0 Mb; *P < *0.01).

### Structure of the cellulose economy.

Taxa designated as strongly ^13^C enriched and cellulolytic (i.e., encoding endoglucanases) represented the greatest proportion of unassembled small subunit (SSU) gene fragments in metagenomes. The most abundant were classified into well-known genera of cellulolytic soil organisms, including *Cellvibrio*, *Herpetosiphon* (*Chloroflexi*), and members of the fungal order *Sordariales* (predominantly *Chaetomium*), as well as lesser-known cellulolytic genera, such as *Devosia* and *Sphingomonas* (Fig. 2). Peptides from these five taxa were also abundant within the total metaproteome (*n*_total_ = 90,557 peptides; 33,765 unique proteins), occupying the following percentages of total peptides: *Rhizobiales*, 6.9%; *Cellvibrionales*, 2.6%; *Sphingomonadales*, 2.6%; *Herpetosiphon*, 1.0%; and *Sordariales*, 0.5%. Endoglucanases were detected in contigs classified into 22 of the 30 genera designated as strongly ^13^C enriched, consistent with their presence in reference genomes (Fig. 2; see Table S3 at https://osf.io/tb3n4/). The 22 genera designated strongly ^13^C enriched and cellulolytic comprised 29% of the total SSU rRNA gene fragments. In contrast, the seven genera designated strongly ^13^C enriched and noncellulolytic (Fig. 2) comprised 2.3% of recovered SSU rRNA reads. The composition of phylobins was in close agreement with prior 16S rRNA gene amplicon data (11) based on gene similarity and taxonomic classification (see Table S3 at https://osf.io/tb3n4/).

A diverse set of endoglucanases was recovered from phylobins, revealing a snapshot of the functional diversity of cellulolytic populations. A total of 430 unique endoglucanase homologs were identified (at a >80% identity threshold) belonging to 40 different carbohydrate-active enzyme (CAZy) families/subfamilies. Eighty-two of these endoglucanases were present within gene clusters that contained a carbohydrate-binding module. A total of 52 peptides in the metaproteome matched endoglucanases; the most abundant was a GH9 from *Cellvibrio* (Table 1). The second and third most abundant endoglucanases in the metaproteome were encoded by fungi (GH131 and GH7). Overall, most endoglucanases in the metaproteome were encoded by fungi (57%), which was disproportionate to the total relative abundance of fungal peptides (1.2%) in the metaproteome.

**TABLE 1:**
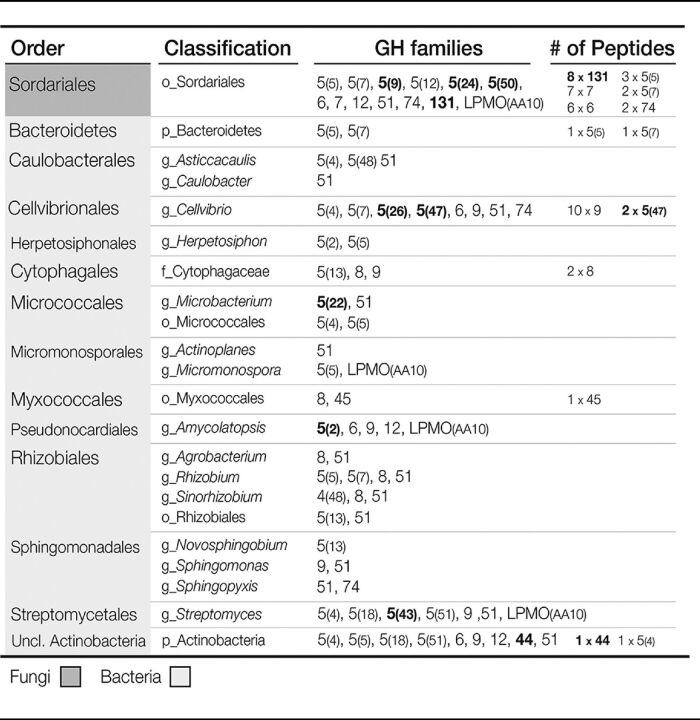
GH and LPMO gene families encoded in cellulolytic phylobins and detected in metaproteome^*a*^

a Diverse glycosyl hydrolase (GH) genes and a single family of lytic polysaccharide monooxygenases (LPMO) were identified in cellulolytic phylobins, many of which matched peptides detected in the metaproteome (the number and type of each peptide is indicated). Taxonomic classifications and GH family are provided for the endoglucanase gene fragments found within each phylobin (lowest LCA classified to the order (o_), family (f_), or genus (g_) level). Taxon-specific endoglucanase families are indicated in bold. Five peptides matched endoglucanase genes not belonging to any phylobins. Uncl., unclassified.

### Evidence for metabolic dependency and competitive exclusion.

**FIG 3:**
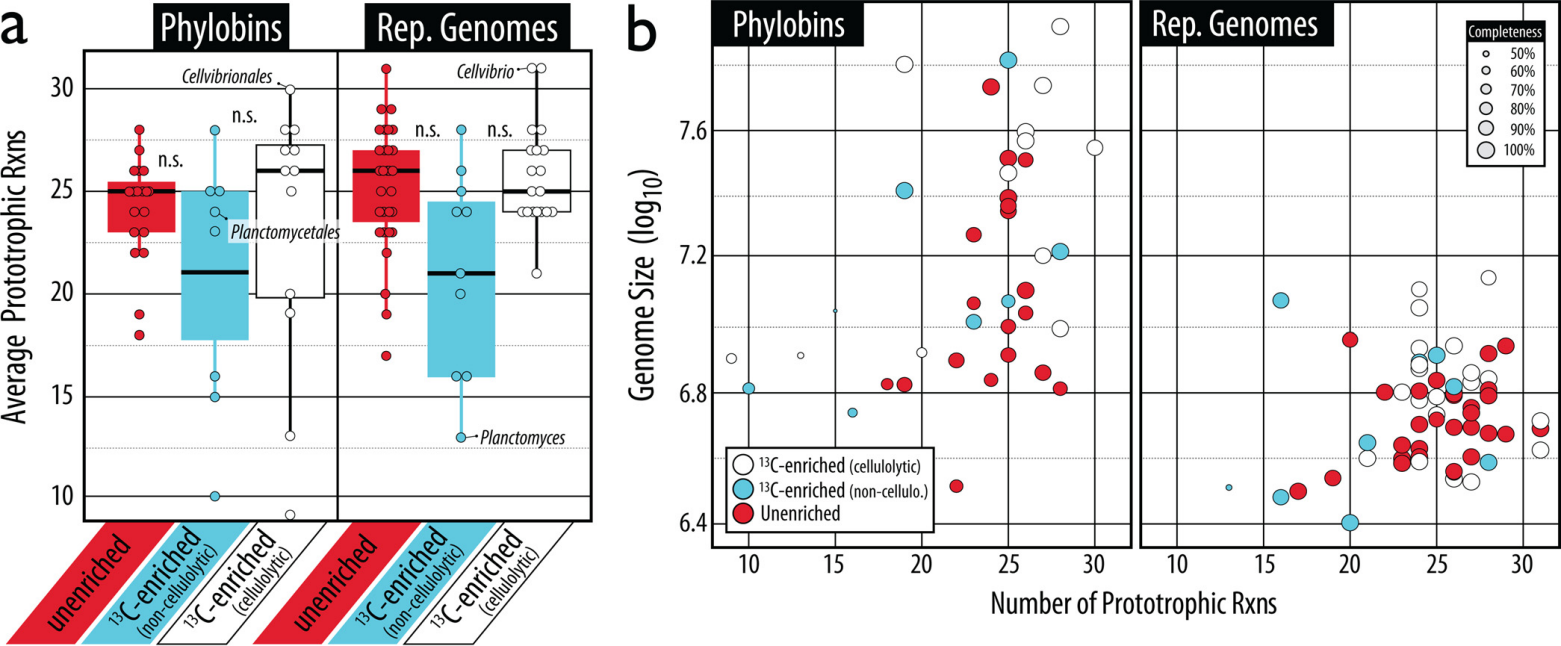
(a) A comparison of the number of complete biosynthetic pathways (prototrophy) revealed that ^13^C-enriched noncellulolytic (blue) phylobins and representative genomes were slightly less prototrophic than those which were either ^13^C-enriched cellulolytic (white) or unenriched (red). On average, these differences were not significant (Kruskal-Wallis; phylobins, *P = *0.6; representative genomes, *P = *0.2), although major populations within each group (*Planctomyces* versus *Cellvibrio*) exhibited consistent differences in accordance with hypotheses. (b) Prototrophy was significantly correlated with genome size for phylobins (*r* = 0.39; *P = *0.01) but not representative genomes (*r* = 0.14; *P = *0.28). A ranking of prototrophy in all phylobins and representative genomes is available in Table S4 at https://osf.io/tb3n4/.

To evaluate potential interactions among ^13^C-labeled taxa, we assessed the degree of auxotrophy (as an indicator of metabolic dependency) and presence of SM-encoding genes (antibiotic-based competition) in phylobins and their representative genomes. No phylobin or genome was fully prototrophic or auxotrophic for all biosynthetic pathways evaluated (*n *= 32), with the average phylobin being auxotrophic for 6 amino acids, 1 cofactor, and 2 vitamins and the average representative genome being auxotrophic for 5 amino acids, 1 cofactor, and 1 vitamin. The extent of auxotrophy did not differ significantly between ^13^C-enriched cellulolytic and ^13^C-enriched noncellulolytic phylobins or representative genomes (Fig. 3) but did differ among dominant taxa within each group.

The most prototrophic representative genomes were *Cellvibrio* (31/32 pathways detected; genome size = 5.2 Mb; designated ^13^C enriched, cellulolytic), *Devosia* (31/32; 4.2 Mb; ^13^C enriched, cellulolytic) and *Leptothrix* (31/32; 4.9 Mb; unenriched, noncellulolytic) (see Table S4 at https://osf.io/tb3n4/). The most auxotrophic representative genomes were *Planctomyces* (13/32; 3.2 Mb; ^13^C enriched, noncellulolytic), *Nannocystis* (16/32; 12.1 Mb; ^13^C enriched, noncellulolytic), and *Vampirovibrio* (16/32; 3.0 Mb; ^13^C enriched, noncellulolytic). These trends were consistent in phylobins, where *Cellvibrionales* (30/32; ranked 1st in terms of biosynthetic capacity among the 38 phylobins examined) and *Rhizobiales* (28/32; ranked 3rd) were among the most prototrophic, while *Planctomycetales* (24/32; 20th), *Vampirovibrionales* (15/32; 34th), *Chthoniobacterales* (10/32; 37th; ^13^C enriched, noncellulolytic), and *Chloroflexales* (9/32; 38th; ^13^C enriched, cellulolytic) were among the most auxotrophic. Overall, representative genomes from the phylum *Actinobacteria* were significantly more auxotrophic than *Proteobacteria* (μ_actino_ = 24.2 versus μ_proteo_ = 25.3; Wilcoxon test, *P = *0.04) driven largely by *Alphaproteobacteria* (μ_alpha_ = 26.2, *P = *0.003; see Fig. S3 at https://osf.io/tb3n4/). This trend was consistent, but not significant, in phylobins (μ_actino_ = 23.8 versus μ_alpha_ = 26.0). Representative genomes for *Actinobacteria* and *Alphaproteobacteria* did not significantly differ in size (μ_actino_ = 5.8 Mb versus μ_alpha_ = 5.3 Mb; Wilcoxon test, *P = *0.46) or completeness (μ = 99.6% in both; *P* = 0.71).

The number of SM genes encoded in ^13^C-enriched cellulolytic phylobins (μ_rep_ = 14.0; μ_PhyBin_ = 40.0) was significantly higher than that in ^13^C-enriched noncellulolytic (μ_rep_ = 5.8; μ_PhyBin_ = 12.4; Wilcoxon test, *P < *0.01) or unenriched (μ_rep_ = 4.8; μ_PhyBin_ = 10.0) phylobins. The trend remained after normalization to total phylobin (2.2 read counts per million [rcpm] versus 1.9 rcpm and 1.3 rcpm) or reference genome size (1.6 rcpm versus 1.0 rcpm and 1.0 rcpm). The genomes encoding the greatest number of SMs were *Sporocytophaga*, several *Actinobacteria* (*Streptomyces*, *Lentzea*, *Dactylosporangium*, and *Kitasatospora*) ,and *Cellvibrio* (see Table S4 at https://osf.io/tb3n4/). Genes encoding type 1 polyketide synthases were consistently more abundant in ^13^C-enriched cellulolytic taxa than the other two groups (see Fig. S4 at https://osf.io/tb3n4/). Nonribosomal peptide synthetases and bacteriocins were more frequently encoded in both ^13^C-enriched groups, but only peptides matching cellulolytic taxa were present in the metaproteome (see Fig. S4 at https://osf.io/tb3n4/). The metaproteome was dominated by terpene synthases from *Actinobacteria*, bacteriocins from *Cellvibrio*, and nonribosomal peptide synthetases from *Sordariales*. In contrast to trends in auxotrophy, representative genomes of ^13^C-enriched cellulolytic *Actinobacteria* encoded significantly higher numbers of SMs (μ = 20.0; *n* = 6; *P = *0.03) than the cellulolytic *Alphaproteobacteria* (μ = 5.3; *n* = 6).

The orders *Planctomycetales* and *Sphingomonadales* were represented by phylobins that were weakly ^13^C enriched, alongside those that were strongly ^13^C enriched and unenriched (Fig. 2). Only the strongly ^13^C-enriched *Sphingomonadales* phylobin encoded endoglucanases and more SMs (predominantly bacteriocins) than the weakly ^13^C-enriched and unenriched phylobins (2.1 rcpm, 0.4 rcpm, and 1.3 rcpm, respectively). Both ^13^C-enriched *Sphingomonadales* phylobins shared the same pattern of auxotrophy (see Fig. S5 at https://osf.io/tb3n4/). No *Planctomycetales* phylobins encoded endoglucanase nor a substantial number of SMs.

### Comparison of cellulolytic and hydrolytic potential.

**FIG 4:**
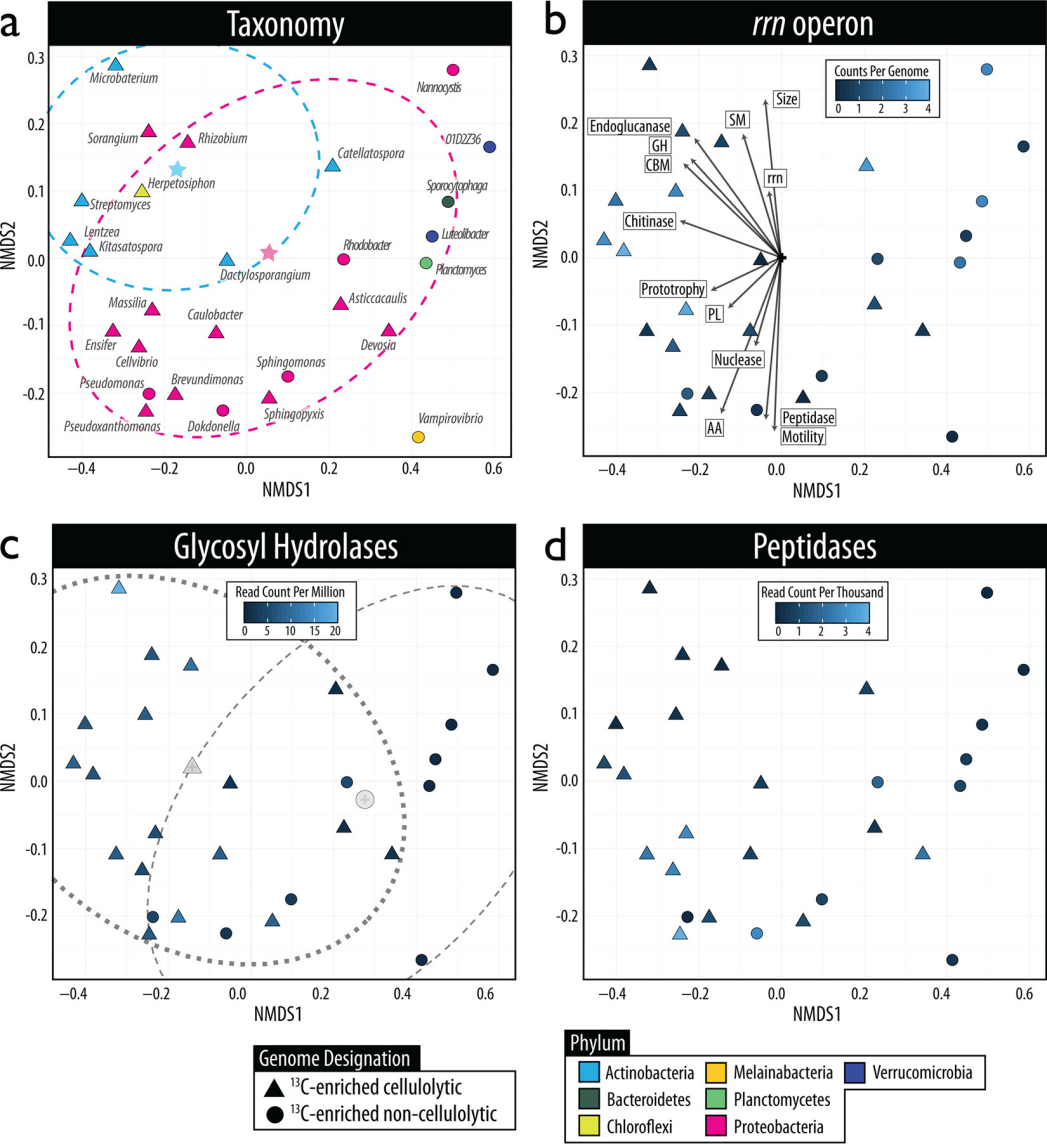
The functional gene contents of representative genomes were compared by NMDS using the Bray-Curtis dissimilarity of gene abundances normalized to genome size. Most variation among representative genomes was attributable to carbohydrate active enzyme content (63% of NMDS1) (see Table S5 at https://osf.io/tb3n4/). Four panels showing the same ordination are colored according to the taxonomic classification at the phylum level (a), the *rrn* operon copy number (b), the abundance of glycosyl hydrolases (c), and peptidase abundance (d). Genomes formed clusters according to cellulolytic potential (analysis of similarities [ANOSIM], *R* = 0.498, *P < *0.001) with the centroid of each group displayed in panel c. Genomes loosely clustered by phylogenetic differences between *Actinobacteria* and *Proteobacteria*, although lacking statistical support (ANOSIM, *R* = 0.1, *P = *0.2), with the centroid (star symbol) for each shown in panel a. In panel b, functional gene data were fitted to the ordination, with the arrow length proportional to the correlation between each variable and ordination axes. Abbreviations: GH, glycosyl hydrolase; SM, secondary metabolite gene cluster; CBM, carbohydrate-binding module; rrn, ribosomal operon; PL, polysaccharide lyase; AA, auxiliary activity; size, genome size.

The functional gene content of representative genomes accounted for substantial variation in enrichment status (Fig. 4). The trend was driven primarily by the relative abundance of glycosyl hydrolases (GH), which were 1.5- to 3-fold higher (after normalization for genome size) in ^13^C-enriched cellulolytic phylobins and corresponding reference genomes, respectively. This trend was evident in all gene families associated with lignocellulose degradation (GH, carbohydrate-binding modules [CBMs], auxiliary activity enzymes [AA], and polysaccharide lyases [PL]), which collectively explained 63% of the variation in community functional composition along nonparametric multidimensional scaling axis 1 (NMDS1) (Fig. 4a; see Table S5a at https://osf.io/tb3n4/). Genomes were also separated along the secondary axis (NMDS2) based on genome size and peptidase and motility gene content, which explained 16.3, 16%, and 19% of variation, respectively (see Table S5a). The degree of auxotrophy did not correlate with variation in functional gene content in either representative genomes or phylobins (see Table S5b at https://osf.io/tb3n4/). In addition, the relative abundance of biomass-degrading enzymes (e.g., peptidases and nucleases) did not differ with respect to degree of ^13^C enrichment or cellulolytic capacity, either in phylobins or representative genomes. In contrast, broad differences in functional gene categories were observed between *Actinobacteria* and *Proteobacteria* (Fig. 4a).

### Temporal dynamics in cellulose economy.

**FIG 5:**
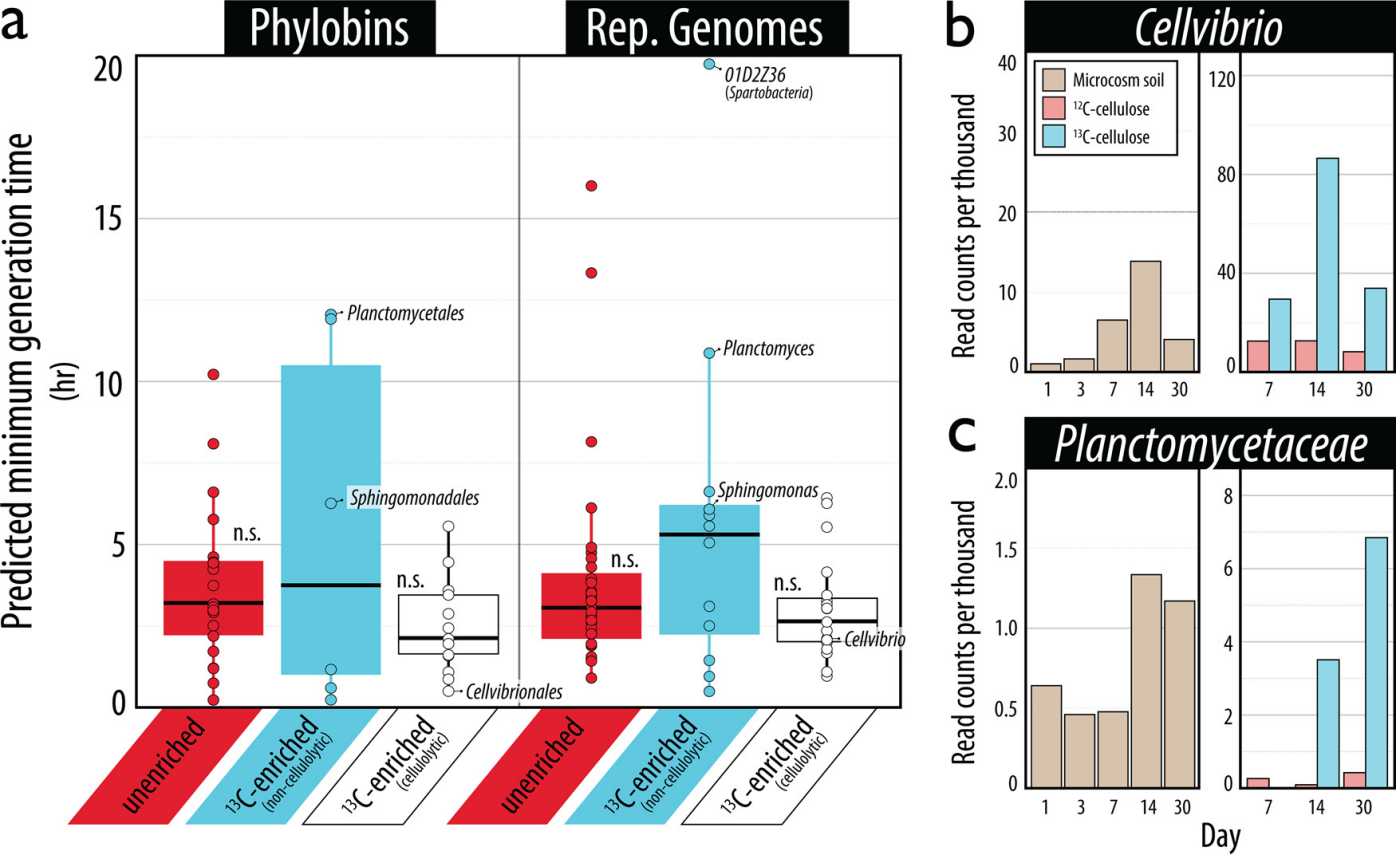
Genome-based predicted generation times of taxa (a) corresponded with temporal patterns in relative abundance in soil microcosms amended with cellulose (b and c), where ^13^C-enriched cellulolytic phylobins and representative genomes tended to have higher growth rates than noncellulolytic phylobins and their representatives. In panel a, the differences in genome-based predictions of generation time were, on average, not significant (Kruskal-Wallis, *P*_phybin_ = 0.33 and *P*_rep_ = 0.52). However, the most abundant ^13^C-enriched cellulolytic and noncellulolytic taxa, *Cellvibrio* and *Planctomycetaceae*, respectively, exhibited characteristics of faster (b) and slower (c) growth, consistent with expectations of the degree of their metabolic dependency. Several other major taxa, including *Devosia*, *Sphingomonas*, and members of the *Verrucomicrobia*, exhibited similar trends (see Fig. S6 at https://osf.io/tb3n4/). In panels b and c, each panel is divided into two data sets: one corresponding to the relative abundance of bacterial populations in whole DNA extract from soil amended with cellulose, and the other corresponding to the relative abundance in ^13^C-enriched DNA pools from soils amended with ^12^C natural abundance or ^13^C-labeled cellulose. The *y* axis (read counts per thousand) corresponds to the average relative abundance of heavy CsCl gradient fractions (F_2_ – F_10_). A complete ranking of predicted generation times for phylobins and representative genomes is available in Table S4 at https://osf.io/tb3n4/.

Early and late-stage colonizers of cellulose were identified according to genome-based predictions of growth rate (see Table S4 at https://osf.io/tb3n4/). Taxa designated as ^13^C enriched and cellulolytic were predicted to have faster generation times based on phylobins (3.0 h) and representative genomes (3.1 h) than ^13^C-enriched noncellulolytic taxa (5.7 h and 5.6 h, respectively) (Fig. 5a), although these differences were not significant (Kruskal-Wallis, *P*_phybin_ = 0.33 and *P*_rep_ = 0.52). The ^13^C-enriched noncellulolytic taxa exhibited a bimodal distribution of predicted growth rate (Fig. 5a), and the set of genomes with slower generation times (generation time > 5 h) was significantly more auxotrophic (μ_slow_ = 15.8/32 prototrophies) than taxa with faster predicted generation times (<3 h; μ_fast_ = 23.8/32; Wilcoxon test, *P = *0.05).

The genome-based characterizations of early and late-stage colonizers were consistent with temporal patterns of taxa observed in time course amplicon sequencing data in a companion study (11). The highly prototrophic taxa *Cellvibrio* and *Devosia* increased in relative abundance earliest, peaking in ^13^C enrichment at days 7 to 14 and declining by day 30 (Fig. 5b; see Fig. S6a at https://osf.io/tb3n4/). *Chaetomium* populations were also early colonizers, showing ^13^C enrichment by day 7 (see Fig. S7 at https://osf.io/tb3n4/). In contrast, the relative abundance of *Actinobacteria* was less dynamic, and these organisms tended to become labeled on, or after, day 14. Taxa predicted to be the most slowly growing, and identified as ^13^C enriched, noncellulolytic, and highly auxotrophic (*Planctomyces*, *Sphingomonas*, and members of *Verrucomicrobia*), began to increase in relative abundance only after day 14 and were maximally ^13^C enriched on day 30 (Fig. 5c; see Fig. S6c at https://osf.io/tb3n4/).

### Surface adhesion and surface motility.

Phylobins from cellulolytic taxa were more likely to encode the capacity for surface adhesion and/or surface motility than other groups, including twitch motility, pilus systems, and fimbriae (see Fig. S8a at https://osf.io/tb3n4/). Surface attachment proteins were abundant in reference genomes from ^13^C-enriched taxa (both cellulolytic and noncellulolytic) and in phylobins classified as *Rhizobiales* and *Caulobacterales* (Fig. S8b). Adhesion proteins used in gliding motility (*aglZ* and *sprB*) were present in reference genomes of cellulolytic taxa but absent from phylobins.

## DISCUSSION

We performed comparative genomics using metagenomic SIP data to test hypotheses about ecogenomic traits occurring in cellulolytic and noncellulolytic microorganisms participating in the cellulose economy. Taxa identified as ^13^C enriched and cellulolytic had larger genomes and a greater number of genes encoding CAZymes, secondary metabolites, surface motility, or surface attachment and tended to have faster generation times than ^13^C-enriched noncellulolytic and unenriched taxa. This evidence supports our hypothesis that the fate of cellulose carbon is mediated by ecological trade-offs between cellulolytic and noncellulolytic taxa. Furthermore, ^13^C-enriched cellulolytic taxa encoded diverse endoglucanases, representing 39 different subfamilies, but no single taxon encoded more than a third of these enzymes, supporting the importance of synergistic interactions among degraders. Auxotrophy was common among both ^13^C-labeled cellulolytic and noncellulolytic taxa, indicating that most taxa acquire essential metabolites from other community members. The average phylobin was auxotrophic for 9 of 32 pathways evaluated, although the highest levels of auxotrophy occurred among noncellulolytic ^13^C-labeled taxa.

The two most prominent ^13^C-labeled cellulolytic taxa, *Cellvibrio* and *Chaetomium*, are commonly abundant in soil from agroecosystems. Both dominated access to cellulosic C in previous SIP cellulose studies of agricultural soils (12, 17) and are favored by tillage (40–43), while *Chaetomium* populations are early successionists in clear-cut forest soil (18). The most abundant endoglucanase in our metaproteome, a GH9 from *Cellvibrio*, was homologous to the main cellulase found in worm castings from agricultural soil (NCBI accession no. ACY24809) (44). The predominance of these ruderal taxa, which we found to be fast growing and self-sufficient (i.e., prototrophic), likely reflects the frequent soil disturbances in agroecosystems. Therefore, the ecological attributes of the cellulose economy we observed may be characteristic of managed agricultural lands. The use of sieving to prepare our soil microcosm might have favored disturbance-adapted organisms. However, in a similar SIP-cellulose study, where soils from different ecosystems were subject to the same microcosm conditions, *Cellvibrio* populations were specific to and dominant in agricultural soil (12). It remains to be seen how the composition and relative importance of the ecological classes we observed differ in less frequently disturbed soils.

### Ecological classes of the cellulose economy.

Our results demonstrated that access to cellulosic carbon is mediated by trade-offs related to the capacity to produce carbohydrate-active enzymes and antibiotics, biosynthetic capacity (i.e., prototrophy), growth rate, and adaptation to colonize surfaces. Our results revealed at least three broad ecologically coherent classes which accessed ^13^C from cellulose: (i) fast-growing, biosynthetically competent cellulolytic taxa (e.g., *Cellvibrio*, *Chaetomium*, and *Devosia*), (ii) slower growing, metabolically dependent (more auxotrophic), cellulolytic taxa (e.g., *Actinobacteria* and *Herpetosiphon*), and (iii) slower growing, metabolically dependent (highly auxotrophic), noncellulolytic taxa (e.g., *Planctomycetales*, *Verrucomicrobia*, and *Vampirovibrionales*). Certainly, a wide range of adaptive traits will affect access to cellulosic carbon during decomposition, but these classes provided a useful framework for grouping the major ecogenomic traits we observed. We also propose that these classes may reflect the broader underlying ecological characteristics of microbial consortia involved in decomposing various forms of particulate organic matter in soil.

### Independent primary degraders.

Bacteria in the class of independent primary degraders are first to colonize cellulosic materials based on their cellulolytic competency, self-sufficiency, and rapid growth. On average, the phylobins and representative genomes of ^13^C-enriched cellulolytic taxa were more prototrophic and had lower minimum generation times than their ^13^C-enriched noncellulolytic counterparts, although these results were statistically insignificant due to the phylogenetic and ecological diversity within groups. *Cellvibrio* and *Devosia* were among the most enriched taxa in the [^13^C]DNA pool (1st and 4th, respectively) and were the two most prototrophic of any genome or phylobin examined. In a companion study, *Cellvibrio* and *Devosia* populations peaked earlier than any other ^13^C-enriched taxa and were in decline as dependent taxa increased in relative abundance (11). *Chaetomium* exhibited similar early ^13^C enrichment, occupying upwards of 20% of the [^13^C]DNA pool by day 7 in a second companion study at the same field site (45). Our method for predicting prototrophy was not validated for eukaryotic genomes; yet, species of *Chaetomium* are prototrophic—growing on cellulose in minimal medium without the addition of amino acids or cofactors (46). The rapid growth and self-sufficiency of *Cellvibrio* and *Chaetomium* were coupled with a strategy of competitive exclusion via the production of antibiotics such as bacteriocin, likely a cellvibriocin (47), and fungicides (48–50). Notably, the early dominance of *Cellvibrio* and *Chaetomium* populations and later shift to *Actinobacteria* were reported in a separate SIP-cellulose study in agricultural soil (17). We expect the competitive nature of these early colonizers and their metabolic by-products to influence the ability of interdependent primary degraders and noncellulolytic taxa to access C derived from cellulose degradation.

### Interdependent primary degraders.

Bacteria in the second class, i.e., interdependent primary degraders, primarily *Actinobacteria* but also *Herpetosiphon* (*Chloroflexi*), were cellulolytic but exhibited higher levels of auxotrophy and SM production than early colonists. Populations of *Actinobacteria* lagged in comparison to *Cellvibrio*, with the first signs of ^13^C labeling appearing at day 14 with inconsistent changes in relative abundance over time (11). These trends suggest a greater dependency on external nutrient sources, affected by top down (i.e., mortality-driven) or bottom up (i.e., nutrient limitation because of competition for nutrients) controls, and may also reflect lower growth rates. *Actinobacteria* encoded and produced the greatest number of SMs and SM peptides. These were primarily terpenoids, which can function in interspecific signaling in soil, potentially facilitating mutualistic interactions upon which *Actinobacteria* depend (51). The potential benefit of interdependency, as a primary degrader, was apparent in the consistent auxotrophy of *Actinobacteria* for four of the costliest nonaromatic amino acids to synthesize, namely, the branched-chain acids isoleucine (ranked 1st) and leucine (2nd), the sulfur-containing methionine (3rd), and the amino-group-containing lysine (4th) (52, 53). Auxotrophy for branched-chain amino acids is a signature of genome reduction and dependency in other ubiquitous soil bacteria (54). We hypothesize that the fitness of interdependent primary degraders depends on community interactions, facilitated by their cellulolytic capacity and SM production.

### Secondary consumers: mutualists, opportunists, and parasites.

The third class, that of secondary consumers, was defined by characteristics of their dependency on the metabolism of primary degraders, indicating that these taxa are secondary consumers of cellulosic carbon. Members of this group all possessed several signatures of metabolic dependency, which included high levels of auxotrophy, the lack of necessary genes for cellulose degradation, and late-stage and low levels of ^13^C enrichment. The most prominent secondary consumers were members of *Planctomycetales*, *Vampirovibrionales*, and *Verrucomicrobia* (*Luteolibacter*, “*Candidatus* Xiphinematobacter,” and 01D2Z36). These taxa all reached maximal relative abundance only after independent primary degraders had become enriched (*Cellvibrio*, *Devosia*, and *Chaetomium*) and remained abundant even after their decline. This pattern suggests a dependence on products of community metabolism either through cometabolism, the consumption of metabolic by-products, or the consumption of macromolecules released during the turnover of microbial biomass. The consequence of these dependencies (i.e., mutualistic versus antagonistic, etc.) could not be determined from our data, but previous research indicates that these taxa encompass a range of symbiotic relationships, including mutualism, opportunism, and parasitism (MOP).

*Planctomyces* are commonly found to colonize the surfaces of marine algae and to metabolize polysaccharides but not cellulose (55–57). They purportedly assimilate oligosaccharides into their cells, indicating the ability to scavenge higher-molecular-weight degradation by-products (13, 58–60). The capacity of *Planctomyces* to attach to surfaces with holdfast and their distinct tolerance to a range of antibiotics would advantage an opportunistic lifestyle, particularly among antibiotic-producing primary degraders (61–63). The cultured representatives for the two other highly auxotrophic ^13^C-enriched noncellulolytic taxa are obligate symbionts, namely, *Vampirovibrio* and “*Candidatus* Xiphinematobacter.” The former are algal parasites that encode a range of GHs (64) but lack endoglucanases, and the latter can be endobionts of nematodes and are abundant in forest litter, cellulose-degrading consortia, or in association with *Basidiomycota* (65–68). “*Candidatus* Xiphinematobacter” and *Planctomyces* have been previously identified as part of the cellulose economy in agricultural soil using SIP, with the former hypothesized to be a late-stage, mutualistic cellulosic C consumer (10, 15).

Differentiating between an opportunistic or mutualistic relationship among primary degraders and noncellulolytic taxa presents a challenge where lignocellulose is being decomposed. The degradation of other plant biomass by noncellulolytic taxa can benefit primary degraders, supported by the fact that secondary consumers possess the capacity to degrade other carbohydrate polymers, like *Vampirovibrio* and *Planctomyces* (57, 64). One set of phylobins provided evidence for what could be considered opportunistic “cheating” (24). Phylobins from *Sphingomonadales* differed in terms of weak and strong ^13^C enrichment yet shared the same pattern of auxotrophy. The strongly enriched phylobins encoded several endoglucanases and bacteriocins, while the equally sized, weakly enriched phylobins lacked these capabilities. While we cannot rule out some form of mutualism, the weakly ^13^C-enriched phylobin did not encode any unique CAZyme families associated with hemicellulose or pectin degradation. These data suggest that the strongly labeled cellulolytic strain is degrading [^13^C]cellulose extracellularly and the weakly ^13^C-enriched strain can access degradation products as well as other sources of unlabeled carbon present in soil. The capacity of *Sphingomonas* to degrade cellulose through the activity of extracellular enzymes has been reported (69, 70).

### Role of surface ecology.

Several major populations of microbes that accessed ^13^C from cellulose were capable of surface adherence and/or surface motility. Genes encoding surface attachment were present in phylobins, or have been previously reported (Fig. 2), in *Rhizobiaceae* (*Ensifer*/*Sinorhizobium*, *Rhizobium*, and *Agrobacterium*), *Hyphomicrobiaceae* (*Devosia*), *Sphingomonadaceae* (*Sphingomonas*), and *Caulobacteraceae* (*Asticcacaulis*, *Brevundimonas*, and *Caulobacter*), as well as in *Pseudoxanthomonas* and *Planctomycetaceae* (*Planctomyces* and *Rhodopirellula*) (71–73). Each of these genera, except for those in *Planctomycetaceae*, are represented by isolates capable of degrading cellulose (74–81). For these organisms, attachment would provide preferential access to the extracellular by-products of cellulose degradation. This phenomenon is exemplified by the abundance of sugar transporters located on the stalk used by *Caulobacter* to adhere to surfaces (82, 83). Attachment may also facilitate cooperation to crowd out competitors from accessing resources, as observed in the social behavior of *Caulobacter* during xylan degradation (84) or in the coordination of extracellular degradative processes by the surface-gliding bacteria *Herpetosiphon* and *Sorangium* (85, 86). Social interactions and cell surface density are critical determinants of the rate and efficiency of decomposition of particulate carbon (22). Thus, the dynamics of surface attachment have ramifications for microbial ecology and evolution, as observed in the rumen (87, 88) and aquatic niches (22, 31, 89), which have not yet been studied in soil or in relation to biogeochemical cycling. For example, our observations suggest that the universal priming effect induced by cellulose (90) might result from promoting the growth of surface-adapted taxa which can subsequently gain access to insoluble, less bioavailable C pools, such as particulate and surface-associated organic matter (91).

### Diversity at the subgenus level in the cellulose economy.

Deep shotgun metagenomics provided a comprehensive set of genomes from taxa present in the cellulose economy but was ineffective at resolving the genomes of closely related species. Phylobins were comprised of large pangenomes, which limited our ability to test for adaptive gene loss among closely related species, known to be important in the evolution of metabolic dependencies (24, 92). The recovery of large single-genus phylobins for *Myxococcales* (*Sorangium*), *Cellvibrionales* (*Cellvibrio*), *Planctomycetales* (*Planctomyces*), and *Micrococcales* (*Microbacterium*) provided evidence of sizeable pangenomic genetic diversity, which might reflect niche partitioning among close relatives. However, the degree of ^13^C enrichment within these single-genus phylobins did not differ, except for *Planctomycetales* and *Sphingomonadales* (i.e., “weak” versus “strong” phylobins). We conclude that few differences in the capacity to access cellulosic carbon was evident among closely related populations.

### Conclusions.

The taxonomic composition of ^13^C-labeled populations was consistent with past SIP-cellulose experiments, represented by populations of *Sordariales* (*Chaetomiaceae*), *Actinobacteria* (*Microbacteriaceae*, *Streptomycetaceae*, and *Micrococcaceae*), *Alphaproteobacteria* (*Rhizobiaceae*, *Caulobacteraceae*, and *Sphingomonadaceae*), Deltaproteobacteria (*Polyangiaceae*), *Gammaproteobacteria* (*Cellvibrionaceae*), *Bacteroidetes* (*Cytophagaceae* and *Sphingobacteriaceae*), *Planctomyces* (*Planctomycetaceae* and *Pirellulaceae*), and *Verrucomicrobia* (*Verrucomicrobiaceae*, *Opitutaceae*, and *Chthoniobacteraceae*), although missing *Betaproteobacteria* (9–18). Yet, for the first time, comparative genomics was used to reveal the ecogenomic traits of these taxa and to deepen understanding of the ecological strategies they employ to gain access to cellulosic C. We identified self-sufficient cellulolytic bacteria and fungi (e.g., *Cellvibrio* and *Chaetomium*) that sought to restrict access via competitive exclusion and other more interdependent cellulolytic bacteria (e.g., *Actinobacteria* and *Herpetosiphon*), whose fitness depended on the metabolic by-products of the community. A third class of noncellulolytic taxa that accessed cellulosic C (e.g., *Planctomycetes*, *Vampirovibrio*, *Verrucomicrobia*) were characterized by dependency on community resources that encompassed mutualistic, opportunistic, and parasitic interactions, which have not yet been fully described due to challenges in cultivability (64). Overall, our findings attest to the existence of an economy underscored by ecological interactions and novel physiological adaptations that impact the degradation of cellulose in soil. These findings improve our capacity to interpret and quantify the effect of community structure and function on decomposition and carbon cycling.

## MATERIALS AND METHODS

### Sample description and recovery of ^13^C-enriched DNA.

DNA-SIP was performed using an agricultural soil incubated with ^13^C-labeled cellulose for 30 days to capture the labeling of primary and secondary populations, as performed previously (11). In brief, a microcosm was prepared with 10 g of soil from a tilled agricultural field under organic management in Penn Yan, New York, as previously described (93). The soil was sieved (2 mm), homogenized, wetted to and maintained at 50% water holding capacity, corresponding to 70% water filled pore space (94), and preincubated for 2 weeks prior to initiation of the experiment. After the initial soil respiration subsided, an amendment designed to approximate the composition of plant biomass was added at a total of 2.9 mg C g^−1^ soil (dry weight). By weight, the mixture was comprised of 38% ^13^C-labeled bacterial cellulose (99 atom% ^13^C), 23% lignin alkali, 20% xylose, 3% arabinose, 1% galactose, 1% glucose, 0.5% mannose, 10.6% amino acids, and 2.9% Murashige Skoog basal salt mixture (11). Bacterial cellulose was harvested from Gluconoacetobacter xylinus grown on 99 atom% [^13^C]glucose as the sole carbon source as previously described (11). After incubation, extracted DNA was subjected to CsCl density gradient centrifugation and fractionated into 35 100-μl aliquots. Shotgun metagenomes were prepared from eight gradient fractions, starting at a buoyant density (BD) of 1.749 g ml^−1^ (F_6_) and continuing to a BD of 1.717 g ml^−1^ (F_13_). A schematic overview of the methods used in this study is presented in Fig. S1 at https://osf.io/tb3n4/. The 16S rRNA gene and ITS1 region amplicon data from this DNA-SIP experiment (11) and a companion study (45) are available at the NCBI under BioProject no. PRJNA317227 and PRJNA589050, respectively.

### DNA and peptide sequencing.

Shotgun metagenomes were generated by multiplexing DNA from each gradient fraction using the Nextera XT library preparation kit and then sequenced using three lanes of the Illumina HiSeq 2500 (150 bp, paired end). A subsequent round of sequencing was performed on each gradient fraction using a single lane of MiSeq (250 bp, paired end) using a library prepared with the Illumina Nextera XT DNA library prep kit (product number FC-131-1024; Illumina). The raw sequencing data are archived in the European Nucleotide Archive (BioProject no. PRJEB23737). A full description of protein extraction, purification, digestion, mass spectroscopy, and peptide annotation is available in the supplementary methods at https://osf.io/tb3n4/. In brief, protein was extracted from 5 g of soil with the NoviPure soil protein kit (Qiagen), initially separated and massed using a Waters nano-Acquity M-class dual pumping UPLC system (Milford, MA) and a Q-Exactive HF mass spectrometer (Thermo Scientific, San Jose, CA). Twenty-four fractions were subsequently submitted for liquid chromatography-tandem mass spectrometry (LC-MS/MS) analysis using an LTQ Orbitrap Velos mass spectrometer (ThermoFisher, Waltham, MA). Peptides were identified from LC-MS/MS data using predicted protein sequences from the metagenome and filtered with a false discovery rate cutoff of 1%.

### Assembly and classification of SSU RNA genes.

Partial 16S and 18S rRNA gene fragments were identified in unassembled reads to estimate relative abundances. Fragments were identified using Infernal (v. 1.1.2) (95) and assigned taxonomy using the mothur implementation of the RDP Classifier (96, 97) with the Silva database (silva.nr_v128) as the reference (98). Full-length 16S or 18S rRNA genes were assembled using MATAM (99), also using the Silva database. We manually recovered a full-length 16S rRNA gene for *Vampirovibrio*, which was prevalent in SSU fragments, but not assembled by MATAM (see details in the supplementary methods at https://osf.io/tb3n4/).

### Shotgun metagenome assembly.

Metagenomes for each gradient fraction were composited and assembled using an iterative process to maximize assembly quality (see Fig. S1 and details in the supplementary methods at https://osf.io/tb3n4/). In brief, an initial *de novo* assembly was performed using megahit (v1.1.1-2-g02102e1) (100). Contigs shorter than 2,500 bp were discarded (∼7% of total). Contigs were then classified by the lowest common ancestor (LCA) algorithm implemented by MEGAN (v. 6) (101) based on DIAMOND BLASTX searches (102) against the NCBI nr database (downloaded 3 February 2017). To improve assembly, two additional assemblies were performed on read sets with reduced sequence diversity. This reduction was achieved by segregating unassembled, quality-processed reads by mapping to (i) the LCA taxonomy of the initial assembly, at the rank of order, and (ii) publicly available genomes represented in the full-length 16S rRNA gene library (see Table S6 at https://osf.io/tb3n4/). All assemblies were then merged using MeGAMerge (103) with the latest version of MUMMer (104) (v.4beta) designed for large data sets. Merging improved the assembly statistics as determined by QUAST (105), increasing *N*_50_ from 4,407 to 5,419 (see Table S7 at https://osf.io/tb3n4/).

### Designating ^13^C enrichment of contigs with gradient-resolved SIP.

The relative abundance of every contig across the density gradient (a gradient profile) was determined by calculating average read depth using “jgi summarize bam contig depths” from MetaBAT (106) (v. 2.12.1). The gradient profile of each contig was also simulated with natural abundance of ^13^C (∼1.1 atom% C) to control for variation in GC content by using methods outlined previously (39, 107). A random forest regression model was used to assign a categorical degree of ^13^C enrichment for each contig, namely, “strongly” and “weakly” enriched, “unenriched,” and “bimodal” (i.e., local maxima in both heavy and light portions of the gradient), and “undetermined” (see examples in Fig. S2 at https://osf.io/tb3n4/). The following features were used to build the model: the number of local maxima and minima (and the fraction in which they occurred) and the average read depth in each fraction for observed and simulated gradient profiles. Data from 600 manually curated contigs were used to train the model, which was implemented in the R package caret (108). Model validation was performed on 20% of the training set (see R code in the supplementary data at https://osf.io/tb3n4/).

### Genome binning.

Common tools for reconstructing MAGs, based on kmer frequency and covariance (in our case across the CsCl gradient), were prone to cross-contamination (see supplementary methods at https://osf.io/tb3n4/). In addition, MAGs constructed using standard practices failed to recover genomes from taxa known to be abundant in the metagenome and ^13^C labeled, including *Chaetomium*, *Vampirovibrio*, and members of *Verrucomicrobia* and *Chloroflexales*. Given these limitations, we opted to define a genomic unit based on ^13^C enrichment and LCA classification of contigs, which we term a “phylobin.” Phylobins consisted of contig sets divided by ^13^C enrichment status (i.e., strong, weak. and unenriched) and by the taxonomic rank at the level of order (e.g., strongly enriched *Cellvibrionales*). We justify this approach accordingly: (i) DNA-SIP selectively enriched for a relatively narrow subset of taxa within a given order, and (ii) phylogenetically related organisms with similar enrichment status are likely to share similar genomic and ecological traits. There is no universally appropriate taxonomic rank or phylogenic depth for grouping organisms, since functional traits are conserved at various phylogenetic depths (109). We chose the rank of order as the cutoff because LCA often fails to accurately classify to the species level taxa that are poorly represented in the NCBI nr database. Hence, aggregating at the rank of order decreases the risk of losing genomic information. Prior research has shown that aggregating microbiome data by taxonomic order produced the greatest discriminating power of relevant soil microbial processes (110). The loss of resolution of individual genomes was compensated for by performing all analyses in parallel on reference genomes chosen based on the similarity of full-length SSU rRNA genes recovered in our study (μ_similarity_ = 98%, *n *= 89) or, in some cases, by the only available representative genome for that genus or clade (*n *= 38) (see Table S6 at https://osf.io/tb3n4/).

### Functional gene annotation.

Functional genes were annotated using curated databases relating to genes for motility, adhesion, secondary metabolite (SM) biosynthetic gene clusters, and catabolic enzymes for biomass and cellulose. Fungal genomes were annotated only for SM cellulolytic enzymes. SMs were annotated using the default settings of antiSMASH for bacteria or fungi (v. 4.1.0) (111). Genes involved in cellulolytic activity, namely, those encoding glycosyl hydrolases (GH), endoglucanases (specific GH families), carbohydrate-binding modules (CBMs), polysaccharide lyases (PL), and auxiliary activity enzymes (AA), were annotated using DIAMOND BLASTX searches against a local version of the CAZy database, which includes bacterial and fungal genes (112) (downloaded 20 December 2017). The following endoglucanase-containing glycosyl hydrolase and lytic polysaccharide monooxygenase families were deemed to confer cellulolytic ability: GH5(1), GH5(2), GH5(4), GH5(5), GH5(7), GH5(8), GH5(9), GH5(11), GH5(12), GH5(13), GH5(15), GH5(16), GH5(18), GH5(19), GH5(22), GH5(23), GH5(24), GH5(25), GH5(26), GH5(27), GH5(28), GH5(29), GH5(30), GH5(31), GH5(36), GH5(37), GH5(38), GH5(39), GH5(40), GH5(41), GH5(43), GH5(44), GH5(45), GH5(46), GH5(47), GH5(48), GH5(49), GH5(50), GH5(51), GH5(53), GH6, GH7, GH8, GH9, GH12, GH44, GH45, GH48, GH51, GH61 (now AA9), GH74, GH124, GH131, and AA10. Chitinases were represented by CAZy families GH18 and GH19. Genes encoding nucleases, adhesion (curli and holdfast proteins), and motility were annotated using DIAMOND BLASTX searches against a local version of the NCBI COG database (113) (downloaded 1 May 2018) and, in the case of motility, mapped to KEGG biosynthetic pathways for synthesizing complete motility apparatus (see Table S8 at https://osf.io/tb3n4/). Genes encoding peptidases were annotated using DIAMOND BLASTX searches against a local version of the MEROPS database (114) (downloaded 1 July 2018). The capacity for gliding motility was assessed using canonical genes from three model organisms: the focal adhesion protein in Myxococcus xanthus (AglZ) (115), the SprB and RemA adhesins in Flavobacterium johnsoniae (116, 117), and Gli349 and Gli521 in Mycoplasma mobile (118, 119). Additional adhesion gene families were annotated using compilations of well-characterized proteins, including unipolar polysaccharide synthesis proteins (*upp*) (120) and tight adherence proteins (*tad*) (121). All annotations were based on a sequence identity cutoff of ≥60% across 90% of the full-length gene.

Auxotrophies were determined for each representative genome and phylobin based on genome-enabled metabolic models (GEMs) in KBase (122), according to Henry et al. (123). Briefly, flux balance analysis was performed on GEMs under two growth conditions: on a rich medium containing all potential biomass precursors and on a minimal medium containing only C and essential nutrients. The number of critical enzyme-catalyzed reactions was calculated for each GEM according to the following criteria: (i) the reaction was not involved in central C metabolism, (ii) the reaction was essential and carried flux only under minimal (i.e., not under rich) medium conditions, and (iii) the flux of the reaction was coupled to the production of an essential compound. A genome was considered auxotrophic for a compound if the number of its critical reactions for its biosynthesis was below a compound-specific threshold or if the number of gap-filled critical reactions exceeded a compound-specific threshold. Thresholds were set based on auxotrophy profiles from a dozen well-characterized bacteria in the *Bacteroidetes*, *Firmicutes*, *Alphaproteobacteria*, and *Gammaproteobacteria*.

### Statistical analyses.

Statistics were performed in R v.3.4.2 (124) with the following packages: reshape2, ggplot2, plyr (125–127), Hmisc (128), and phyloseq (129). Nonparametric multidimensional scaling (NMDS) was performed using metaMDS from the R package vegan (130). The relative amount of variation in the primary and secondary NMDS axes explained by functional traits was calculated using the R package relaimpo (131). Pairwise multiple comparisons based on the Kruskal-Wallis test (kruskalmc) were performed using the R package pgirmess (132). Minimum generation times were predicted for all phylobins and representative genomes using growthpred (v.1.07) (133) based on codon usage bias using ribosomal genes (identified by COG ID) as the set of highly expressed genes. All analyses can be reproduced using R scripts, and data are available in the supplementary data package at https://osf.io/tb3n4/.

### Data availability.

All analyses can be reproduced using R scripts, and data are available in the supplementary data package at https://osf.io/tb3n4/. Supplementary data, figures, tables, and methods are hosted at the Open Science Framework at https://osf.io/tb3n4/. The shotgun metagenomes (BioProject no. PRJEB23737), 16S rRNA (no. PRJNA317227), and ITS (no. PRJNA589050) amplicon libraries associated with this study are available through the NCBI.
